# Care partner‐informed meaningful change thresholds for the Clinical Dementia Rating‐Sum of Boxes for trials of early Alzheimer's disease

**DOI:** 10.1002/alz.14050

**Published:** 2024-07-17

**Authors:** Claire J. Lansdall, Edmond Teng, Jerome Chague, Rohan Palanganda, Paul Delmar, Janice Smith, Jeffrey L. Cummings, Fiona McDougall

**Affiliations:** ^1^ Product Development, Patient‐Centered Outcomes Research F. Hoffmann‐La Roche Ltd Basel Switzerland; ^2^ Early Clinical Development Genentech, Inc. South San Francisco California USA; ^3^ Product Development, Data Science F. Hoffmann‐La Roche Ltd Basel Switzerland; ^4^ Product Development, Data Science Roche Products Ltd Welwyn Garden City UK; ^5^ Product Development, Neuroscience Roche Products Ltd Welwyn Garden City UK; ^6^ Chambers‐Grundy Center for Transformative Neuroscience University of Nevada Las Vegas Nevada USA; ^7^ Product Development, Patient‐Centered Outcomes Research Genentech, Inc. South San Francisco California USA

**Keywords:** activities of daily living, Alzheimer's disease, anchor‐based, Caregiver Global Impression of Change – Alzheimer's Disease, Clinical Dementia Rating‐Sum of Boxes, clinical meaningfulness, cognition, function, meaningful change, mild dementia due to Alzheimer's disease, mild cognitive impairment due to Alzheimer's disease, Tauriel

## Abstract

**INTRODUCTION:**

Consensus definitions of meaningful within‐patient change (MWPC) on the Clinical Dementia Rating‐Sum of Boxes (CDR‐SB) are needed. Existing estimates use clinician‐rated anchors in clinically diagnosed Alzheimer's disease (AD) populations. Incorporating the care partner perspective offers important insights, and evaluating biomarker‐confirmed cohorts aligns estimates with ongoing trials.

**METHODS:**

Anchor‐based analyses were conducted to evaluate MWPC on the CDR‐SB in early AD (Tauriel; NCT03289143) using Caregiver Global Impression of Change in memory or daily activities.

**RESULTS:**

Across time points and anchors, mean CDR‐SB changes associated with the “somewhat worse” category ranged from 1.50 to 2.12 in early AD, 1.07 to 2.06 in mild cognitive impairment‐AD, and 1.79 to 2.25 in mild AD.

**DISCUSSION:**

The proposed ranges are appropriate to define meaningful progression on the CDR‐SB in similar cohorts and support the interpretation of treatment benefit through MWPC analyses. Thresholds should be calibrated to the context of use; lower/higher thresholds may be applicable in studies of earlier/later disease over shorter/longer durations.

**Highlights:**

- Within‐patient CDR‐SB change thresholds are provided using caregiver‐rated anchors.
- 1.5 to 2.5 points may be an appropriate range in early AD trials of similar durations.
- Cumulative distribution function plots illustrate the benefit of a given treatment.
- When selecting thresholds, the target population and study design should be considered.

## 1 BACKGROUND

Understanding the clinical meaningfulness of treatment effects for emerging disease‐modifying therapies is essential to support the interpretation of Alzheimer's disease (AD) clinical trials, but it remains unclear what degree of change on frequently used clinical outcome assessments is meaningful for patients and care partners. Meaningful within‐patient change (MWPC) thresholds define a range of score changes on a clinical outcome assessment that reflects a clinically meaningful change in the concept(s) being measured in a specific target population,^1^, ^2^, ^3^ with a specific instrument such as the Clinical Dementia Rating‐Sum of Boxes (CDR‐SB) in AD.^4^ The US Food and Drug Administration Patient‐Focused Drug Development draft guidance documents recommend establishing MWPC thresholds to inform responder/progressor analyses as one approach to support the interpretation of clinical trial results.^2^, ^3^ The application of appropriate MWPC thresholds can provide supportive evidence beyond mean group‐level differences that a novel treatment provides tangible benefits to patients and their care partners. Importantly, these thresholds are intended to be applied at the individual patient level as described, and not to evaluate the meaningfulness of mean differences in change from baseline scores between treatment groups.

One approach to establishing MWPC thresholds is to conduct anchor‐based analyses, which require a suitable “anchor” – often a global measure of change or severity – that correlates sufficiently with the target measure of interest, measuring a similar concept but capturing changes where meaningfulness is directly interpretable or already known.^2^, ^3^, ^5^, ^6^, ^7^ Thresholds for “minimal” MWPC are often based on individuals who fall into categories of a little better/minimal improvement, or a little worse/minimal worsening.^7^ For neurodegenerative diseases such as AD, current therapeutic strategies aim to slow disease progression,^8^, ^9^ and as such, estimating magnitudes of clinically meaningful deterioration rather than improvement provides greater insight into treatment effects. Distribution‐based methods are useful supportive analyses, defining thresholds above which score changes are unlikely to be due to measurement error alone.^3^, ^5^, ^7^ However, purely distribution‐based statistical approaches do not capture whether the change is considered clinically meaningful.

While recent publications provide initial anchor‐based estimates of MWPC on the CDR‐SB based on clinician‐rated anchors of perceived meaningful deterioration,^10^, ^11^ consensus regarding their validity and application is lacking. Incorporating the care partner perspective into estimates of meaningful change is critical; they typically spend more time with the person living with AD than clinicians, observing impaired abilities across a broad range of situations, and may more accurately recognize the level of support needed in daily life. Their perspective is particularly important in AD, where the reliability of patient‐reported outcomes may be impacted by progressive cognitive impairment and lack of fully intact insight.

Previous analyses suggest that MWPC thresholds may differ depending on disease severity,^10^, ^12^ emphasizing the need to calibrate thresholds to the target population. While anchor‐based thresholds established using data from a given cohort will be appropriate for the target sample from which they were derived, their generalizability to other and/or broader populations remains to be determined. To date, thresholds for the CDR‐SB have been derived from observational or interventional trial cohorts of clinically diagnosed AD populations without biomarker confirmation.^10^, ^11^ While these studies have generated similar thresholds, providing some initial converging evidence for their robustness, estimates derived from biomarker‐confirmed cohorts may be more applicable to ongoing clinical trials and may include patients that exhibit faster clinical progression.^13^

For this analysis, MWPC thresholds for the CDR‐SB were derived using the Caregiver Global Impression of Change – Alzheimer's Disease (CaGI‐Alz) in individuals with biomarker‐confirmed early AD (mild cognitive impairment due to AD [MCI‐AD] or mild dementia due to AD [mAD]) using data from an 18‐month clinical trial (NCT03289143). The CaGI‐Alz captures changes in memory and activities of daily living (ADLs) as rated by the care partner, thereby capturing the same concepts of interest as the CDR‐SB. A simulation analysis was conducted to evaluate whether the application of these thresholds could be informative in the context of a positive trial where a treatment effect exists.

## 2 METHODS

### 2.1 Study sample

We analyzed data from the Tauriel study (NCT03289143), an 18‐month, Phase 2, multicenter, randomized, double‐blind, placebo‐controlled, parallel‐group clinical trial that assessed the anti‐tau antibody semorinemab in participants with MCI‐AD or mAD.^14^ Eligible participants were between 50 and 80 years old (inclusive) at the time of screening, met diagnostic criteria for MCI‐AD^15^ or mAD,^16^ and had Mini‐Mental State Examination^17^ scores between 20 and 30 (inclusive), global scores on the Clinical Dementia Rating (CDR)^18^ of 0.5 or 1, Repeatable Battery for the Assessment of Neuropsychological Status^19^ Delayed Memory Index scores of ≤ 85, and evidence of significant cerebral amyloid pathology confirmed by amyloid beta‐protein positron emission tomography scan ([^18^F]florbetaben, [^18^F]florbetapir, [^18^F]flutemetamol, or [^18^F]NAV4694 via visual read) or cerebrospinal fluid (CSF) amyloid beta‐protein(1‐42) levels (≤1000 pg/mL, Elecsys^®^ β‐amyloid [1‐42] CSF immunoassay; Roche Diagnostics, Penzberg, Germany). An informant with frequent and sufficient contact with the patient was required to provide information at clinic visits and sign necessary consent forms. Every effort was made to maintain the same care partner reporter throughout the study. Participants were stratified into MCI‐AD or mAD cohorts per investigator interpretation and central review of respective diagnostic criteria.^15^, ^16^ As no clinical efficacy was observed in any of the semorinemab dose arms relative to the placebo arm,^14^ our analyses used data pooled across all study arms to determine meaningful change thresholds for the CDR‐SB.

### 2.2 Clinical outcome assessments

The CDR is a clinician‐rated assessment of AD severity based on semi‐structured interviews with the participant and a reliable informant (eg, care partner/study partner).^18^ The CDR characterizes the participant's level of cognitive and functional impairment across six domains (memory, orientation, judgment and problem‐solving, community affairs, home and hobbies, and personal care) on a 0 to 3 scale for each domain, with higher scores indicating greater impairment. The CDR‐SB, which was the primary endpoint in the Tauriel study, is calculated by summing the ratings across each of the six domains (range: 0 to 18), with higher scores indicating greater impairment.^4^, ^20^, ^21^

Global impressions of change are commonly used as anchors to determine meaningful change thresholds on target instruments.^3^, ^7^, ^22^ In this study, a study‐specific instrument, the CaGI‐Alz, was used as an exploratory outcome measure to assess change in cognition and function. The CaGI‐Alz is a 7‐point care‐partner‐rated instrument developed to separately assess global change in memory and daily activities via two questions, both of which are analogous to the Clinician's Global Impression of Change scale in terms of their structure/item response options.^23^ Items are rated from “very much worse” to “very much improved” (Figure S1) relative to the start of the study (eg, baseline visit).^22^ The CaGI‐Alz provided two independent anchors of a meaningful decline in memory and ADLs for use in this analysis. The “somewhat worse” category was used as the primary category of interest for establishing MWPC, reflecting a change that is noticeable to the care partner, while the “no change” category represented a key comparator. The CaGI‐Alz items were rater‐administered by a trained clinical or cognitive scale rater.

**RESEARCH IN CONTEXT**

1. **Systematic review**: We reviewed and summarized existing regulatory guidance on the development and validation of clinical outcome assessments and anchor‐based studies to establish meaningful change thresholds for the Clinical Dementia Rating (CDR) in Alzheimer's disease (AD).
2. **Interpretation**: When evaluating clinical trial results in early AD populations over similar durations, meaningful within‐patient progression on the CDR‐Sum of Boxes can be defined using a range of thresholds (1.5 to 2.5 points). Thresholds should be calibrated to the context of use; lower or higher thresholds may be applicable in studies of earlier versus later disease and shorter versus longer duration, respectively. Incorporating the caregiver perspective into estimates of clinically meaningful change provides valuable insights.
3. **Future directions**: This study presents one approach to evaluating the meaningfulness of trial results. Qualitative research may further elucidate which aspects of cognitive and functional change lead care partners to rate the presence or absence of deterioration.

### 2.3 Statistical analyses

All analyses were conducted post hoc on the modified intent‐to‐treat cohort, which included all participants with CDR‐SB data at baseline and at least one post‐baseline time point.^14^ Both anchor‐based and distribution‐based analyses were performed to estimate thresholds for meaningful within‐person change on the CDR‐SB.^3^, ^7^, ^24^ Each participant's change from baseline on the CDR‐SB at Weeks 25, 49, and 73 relative to CaGI‐Alz responses from the corresponding time point was compared. The appropriateness of each candidate anchor for the analyses was determined via Spearman's correlation analyses between change in the target measures and change as rated on the anchor measures. Since a Spearman's rank correlation coefficient strength greater than or equal to 0.3 is considered appropriate for anchor‐based analyses,^7^, ^25^, ^26^ we limited our analyses to those time points where the correlation coefficients between the CaGI‐Alz and change from baseline on the CDR‐SB exceeded this threshold. Empirical cumulative distribution function (eCDF) plots were generated to evaluate the appropriateness of candidate anchors and visually assess their ability to meaningfully differentiate between groups, with a focus on the “no change” and deterioration categories, given the progressive nature of AD.

Distribution‐based analyses were derived from baseline performance on the CDR‐SB and included 0.5 times the standard deviation (SD) of baseline scores and standard error of measurement (SEm).^7^ SEm was derived from the formula SEm = SD × √1−*r*, where SD is the standard deviation of an endpoint score at baseline and *r* is a test–retest reliability estimate.^27^ Because it includes reliability (*r*), this distribution‐based estimate explicitly considers measurement precision and presents average random error unexplained by the measured construct. These supportive analyses establish minimal thresholds of change from a statistical perspective: changes above these distribution‐based thresholds are considered unlikely to be attributable solely to measurement error. Test–retest analyses were conducted to provide reliability estimates for the computation of SEm. The reliability coefficient for SEm was *r* = 0.8.

Simulation analyses were conducted to generate a 30% relative reduction between hypothetical treatment and placebo groups at Weeks 49 and 73; this was done by multiplying the existing placebo results by 0.7, calculating their means and covariances, then using the results to produce a random sample from a normal distribution. Existing placebo arm results were used to simulate 1000 participants’ data (split as 500 on placebo and 500 on treatment) at Weeks 49 and 73. This was then plotted as an eCDF curve, with the actual placebo and combined treatment arm data presented for comparison. An eCDF curve for an effective treatment on the CDR‐SB is shifted to the left of the placebo curve.

## 3 RESULTS

### 3.1 Sample description

The baseline demographics and clinical characteristics of the 422 participants in the modified intent‐to‐treat cohort are described in Table 1. The MCI‐AD (*n* = 148) and mAD (*n* = 247) subgroups were similar in their demographic characteristics; as expected, the mAD subgroup exhibited more severe clinical impairment.

**TABLE 1:** Baseline demographics and clinical characteristics.

	All participants *n* = 422	MCI‐AD *n* = 148	mAD *n* = 274	*p* (MCI‐AD versus mAD)
**Age, mean (SD)**	69.6 (7.0)	69.9 (7.1)	69.4 (7.0)	0.52^a^
**Sex, *n* (%)**				0.55^b^
Female	235 (55.7)	79 (53.4)	156 (56.9)	
Male	187 (44.3)	69 (46.6)	118 (43.1)	
**Race, *n* (%)**				0.87^b^
White	388 (91.9)	137 (92.6)	251 (91.6)	
Other/unknown	34 (8.1)	11 (7.4)	23 (8.4)	
**MMSE, mean (SD)**	23.3 (2.7)	25.0 (2.4)	22.4 (2.4)	<.001^a^
**CDR‐GS, *n* (%)**				<.001^b^
0.5	282 (66.8)	141 (95.3)	141 (51.5)	
1.0	140 (33.2)	7 (4.7)	133 (48.5)	
**CDR‐SB, mean (SD)**	3.9 (1.6)	2.7 (1.0)	4.5 (1.5)	<.001^a^

Abbreviations: CDR‐GS, Clinical Dementia Rating‐Global Score; CDR‐SB, Clinical Dementia Rating‐Sum of Boxes; mAD, mild Alzheimer's disease dementia; MCI‐AD, mild cognitive impairment due to Alzheimer's disease; MMSE, Mini‐Mental State Examination; SD, standard deviation.

^a^ Two‐sample *t* test.

^b^ Pearson's chi‐squared test.

### 3.2 Appropriateness of CaGI‐Alz for anchor‐based analyses

**FIGURE 1:**
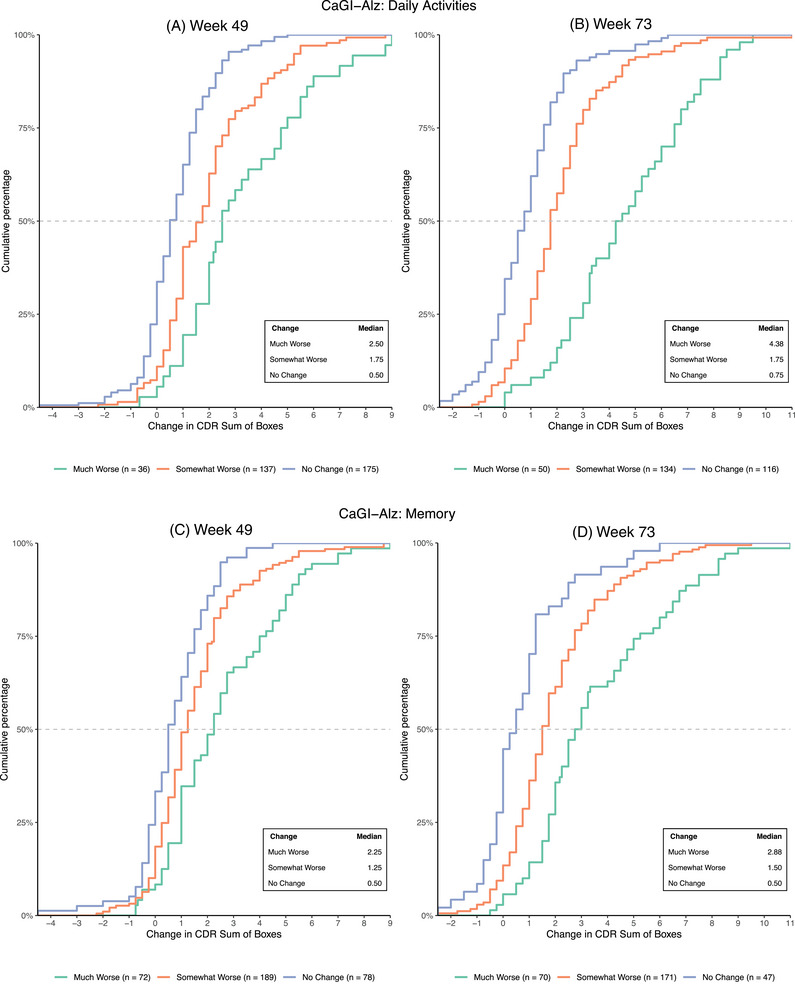
eCDF plots of CDR‐SB change by CaGI‐Alz categories. Note: eCDF plots display the cumulative percentage of individuals experiencing a given change on the CDR‐SB from baseline to Weeks 49 and 73, respectively, grouped into “no change,” “somewhat worse,” or “much worse” on the CaGI‐Alz daily activities (A and B) and CaGI‐Alz memory (C) and (D), respectively. “Improved” category not included in panels of eCDF plots due to low *N* (71), A: Week 49 (*n* = 15, median = 0.75); B: Week 73 (*n* = 10, median = 1.125); C: Week 49 (*n* = 24, median = 0); D: Week 73 (*n* = 22, median = 0.375). CaGI‐Alz, Caregiver Global Impression of Change – Alzheimer's disease; CDR‐SB, Clinical Dementia Rating Scale‐Sum of Boxes; eCDF, empirical cumulative distribution function.

Anchor‐based analyses were used as the primary approach to establish clinically meaningful score changes on the CDR‐SB. Spearman's correlation coefficients at Weeks 25, 49, and 73 for change as rated on the memory and daily activities items (anchors) and change from baseline on the CDR‐SB are shown in Table S1. Analyses focused on the Week 49 and 73 time points, which consistently yielded correlation coefficients > 0.3 between change in the CDR‐SB and the CaGI‐Alz for the combined early AD, MCI‐AD, and mAD samples. eCDF plots (Figure 1, Figures S2,S3) also indicated good separation between anchor categories “no change” and “somewhat worse,” providing additional support for the validity of the anchors.

**FIGURE 2:**
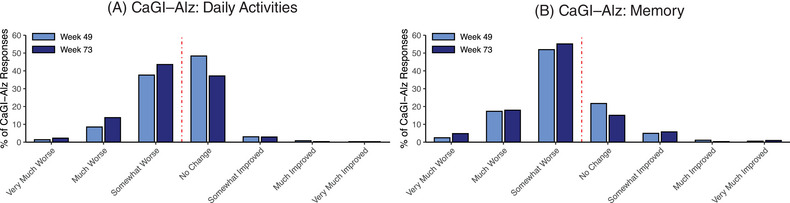
Distribution of CaGI‐Alz daily activities (A) and memory (B) responses. Note: Distributions are provided for the CaGI‐Alz daily activities and CaGI‐Alz memory items, respectively, for Weeks 49 and 73. CaGI‐Alz, Caregiver Global Impression of Change – Alzheimer's disease.

Histograms for the distribution of CaGI‐Alz responses on the memory and daily activities items indicated that most patients were classified into the “no change” and “somewhat worse” anchor categories at the Week 49 and 73 time points (Figure 2). A greater percentage of patients fell into the “somewhat worse” category on the memory anchor relative to the daily activities anchor, and, as expected with disease progression, a greater proportion of participants generally fell into the “somewhat worse” category, while fewer participants fell into the “no change” category on both the memory and daily activities items at Week 73 compared with Week 49.

### 3.3 Anchor‐based estimates

**FIGURE 3:**
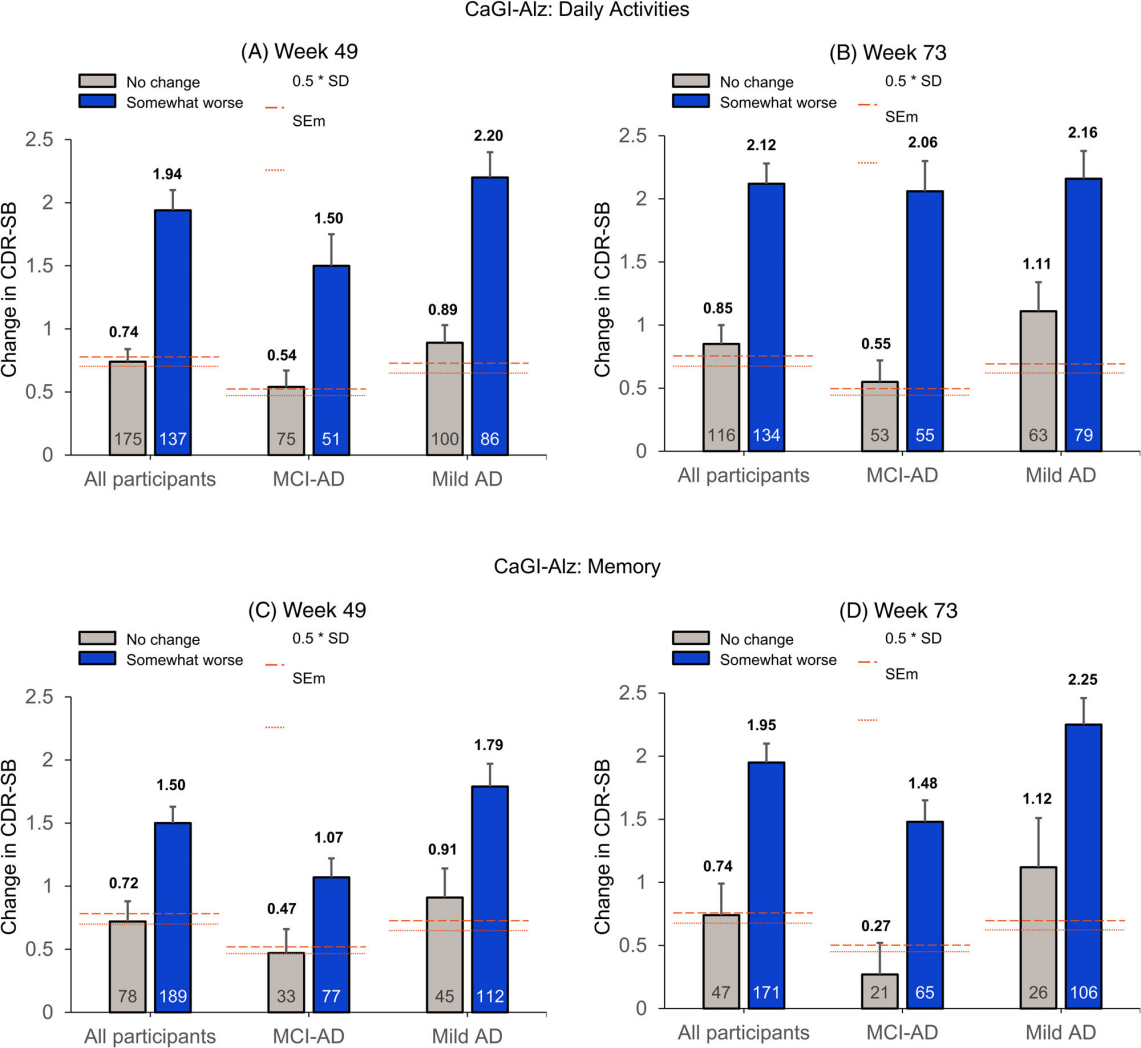
Change in CDR‐SB versus CaGI‐Alz daily activities (A and B) and memory (C and D). Note: Mean change in CDR‐SB is displayed for individuals classified as “no change” or “somewhat worse” on the CaGI‐Alz at Weeks 49 and 73. Groupings include all participants, MCI due to AD, and mild AD. Distribution‐based estimates, including 0.5 * Standard deviation (SD) from baseline and standard error of measurement (SEm), are indicated by the thick and thin red dotted lines, respectively. The value at the bottom of the bar represents the sample size (*n*). The value at the top reflects the mean CDR‐SB change from baseline. SD and SEm values are provided in Table 2. AD, Alzheimer's disease; CaGI‐Alz, Caregiver Global Impression of Change – Alzheimer's disease; CDR‐SB, Clinical Dementia Rating Scale‐Sum of Boxes; MCI, mild cognitive impairment; SD, standard deviation; SEm, standard error of measurement.

At both time points and across both MCI‐AD and mAD subgroups, participants in the “somewhat worse” category for the memory and daily activities anchor had a substantially greater change in CDR‐SB than those in the “no change” category. Mean changes from baseline on the CDR‐SB at Weeks 49 and 73 for participants rated as “no change” or “somewhat worse” on the CaGI‐Alz memory and daily activities items, respectively, are reported in Figure 3 and Table 2. Considering the combined early AD sample, the mean CDR‐SB changes associated with the “somewhat worse” category on the daily activities anchor were 1.94 at Week 49 and 2.12 at Week 73; for the memory anchor, they were 1.50 at Week 49 and 1.95 at Week 73. Slightly greater changes were typically observed at Week 73 compared with Week 49 for the “somewhat worse” category across all groups (early AD, MCI‐AD, and mAD), while no substantial differences were observed for the “no change” group. Mean CDR‐SB change associated with the “no change” group was consistently close to, but generally below, 1 point. Examination of the confidence intervals for the “no change” and “somewhat worse” groups for each anchor by time point and subgroup (Table 2) indicated some overlap for the MCI‐AD Week 49 CaGI‐Alz memory and the mAD Week 73 CaGI‐Alz daily activities, suggesting that these specific estimates should be interpreted with caution.

**TABLE 2:** Summary of anchor‐ and distribution‐based results by group at Weeks 49 and 73, respectively.

			CaGI‐Alz Daily Activities		CaGI‐Alz Memory		Distribution estimates	
Time point	Group	Summary statistics	No change	Somewhat worse	No change	Somewhat worse	0.5 SD	SEm
Week 49	Early AD	*n*	175	137	78	189	0.80	0.71
		Mean	0.74	1.94	0.72	1.50		
		SD	1.33	1.86	1.37	1.73		
		SE	0.11	0.16	0.16	0.13		
		CI	(0.54, 0.94)	(1.62, 2.26)	(0.4, 1.04)	(1.24, 1.76)		
	MCI‐AD	*n*	75	51	33	77	0.52	0.47
		Mean	0.54	1.50	0.47	1.07		
		SD	1.14	1.78	1.10	1.36		
		SE	0.13	0.25	0.19	0.15		
		CI	(0.28, 0.8)	(1, 2)	(0.08, 0.86)	(0.77, 1.37)		
	mAD	*n*	100	86	45	112	0.74	0.66
		Mean	0.89	2.20	0.91	1.79		
		SD	1.44	1.87	1.52	1.90		
		SE	0.14	0.20	0.23	0.18		
		**CI**	(0.61, 1.17)	(1.8, 2.6)	(0.45, 1.37)	(1.43, 2.15)		
Week 73	Early AD	*n*	116	134	47	171	0.78	0.69
		Mean	0.85	2.12	0.74	1.95		
		SD	1.58	1.87	1.69	1.94		
		SE	0.15	0.16	0.25	0.15		
		CI	(0.55, 1.15)	(1.8, 2.44)	(0.24, 1.24)	(1.65, 2.25)		
	MCI‐AD	*n*	53	55	21	65	0.51	0.46
		Mean	0.55	2.06	0.27	1.48		
		SD	1.22	1.78	1.13	1.41		
		SE	0.17	0.24	0.25	0.17		
		CI	(0.21, 0.89)	(1.58, 2.54)	(‐0.25, 0.79)	(1.14, 1.82)		
	mAD	*n*	63	79	26	106	0.72	0.64
		Mean	1.11	2.16	1.12	2.25		
		SD	1.80	1.94	1.97	2.15		
		SE	0.23	0.22	0.39	0.21		
		CI	(0.65, 1.57)	(1.72, 2.6)	(0.32, 1.92)	(1.83, 2.67)		

Abbreviations: AD, Alzheimer's disease; CI, confidence interval; mAD, mild Alzheimer's disease; MCI, mild cognitive impairment; SD, standard deviation; SE, standard error; SEm, standard error of measurement.

In general, slightly greater CDR‐SB changes were associated with the daily activities anchor compared with the memory anchor and for the mAD group compared with the MCI‐AD group. The daily activities anchor indicated no substantial differences across baseline disease severities (ie, between MCI‐AD and mAD) at Week 73, while differences were observed between MCI‐AD and mAD for the daily activitiesanchor at Week 49 and for the memory anchor at both time points.

### 3.4 Distribution‐based analyses

Distribution‐based change thresholds were typically lower than anchor‐based thresholds, falling below 1 point (Table 2), and overlapped with the CDR‐SB changes observed in participants rated as experiencing “no change” on the CaGI‐Alz (Figure 3) across all time points and subgroups.

### 3.5 Simulation analysis

**FIGURE 4:**
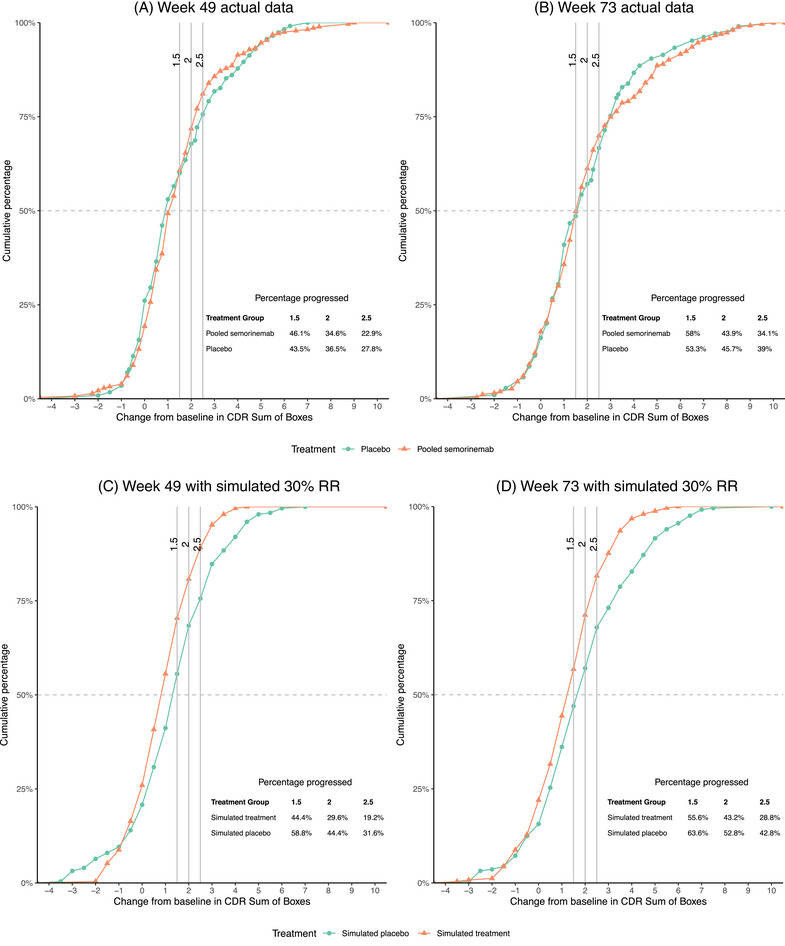
eCDF plots demonstrating cumulative proportion of patients achieving given score change on CDR‐SB by treatment group at Weeks 49 and 73. Note: The proportion of patients progressing by greater than or equal to thresholds of 1.5, 2, and 2.5, by group, are provided in the bottom right table. Panels A and B display actual data from the Tauriel study, comparing treatment (combined semorinemab arms) to placebo. Panels C and D display results from the simulation analysis (simulated treatment and simulated placebo). CDR‐SB, Clinical Dementia Rating‐Sum of Boxes; eCDF, empirical cumulative distribution function; RR, risk ratio.

eCDF plots based on the simulation analysis provided estimates of the cumulative proportion of patients achieving a score change on the CDR‐SB greater than or equal to 1.5, 2, and 2.5 at Weeks 49 and 73 (Figure 4). The proportion of patients across simulated arms that progressed by these thresholds is reported in Figure 4. Comparing the simulated treatment arm with the simulated placebo arm at Week 49, the difference in the proportion of patients progressing by CDR‐SB score change from baseline ≥1.5 was 14.4%, ≥2 was 14.8%, and ≥2.5 was 12.4%. At Week 73, the difference in the proportion of patients progressing by a CDR‐SB score change from baseline ≥1.5 was 7.7%, ≥2 was 9.6%, and ≥2.5 was 14.0%.

## 4 DISCUSSION

This study provides estimates for MWPC on the CDR‐SB based on care partner‐rated anchors of observed change in an individual's memory and ability to perform ADLs, in a biomarker‐confirmed early AD population. These thresholds are useful to support the interpretation of treatment efficacy by assessing individual patient responses (ie, define meaningful within‐patient progression over the course of the trial and report the proportion of “progressors” by treatment arm) and are not intended to interpret the magnitude of between‐group differences (ie, difference in mean change from baseline on drug vs placebo). The MWPC threshold ranges reported in this study are largely aligned with existing estimates from previous anchor‐based analyses in early AD.^10^, ^11^ The convergence of threshold ranges across studies, despite differences in study populations (observational vs clinical trial), anchor types (change vs severity), and, importantly, anchor raters (clinician vs care partner), provides confidence in their robustness and applicability to early AD populations.

Considering the CDR‐SB changes associated with the “somewhat worse” category on the CaGI‐Alz daily activities anchor across time points, 1.5 to 2.5 points is an appropriate range of MWPC thresholds for clinical trials of similar duration in early AD that are biomarker‐confirmed and enriched for faster progression based on inclusion criteria.^11^, ^14^, ^28^We align the recommended thresholds primarily with the daily activities anchor for two reasons. First, the daily activities anchor correlation with the CDR‐SB was slightly higher across all time points, in line with previous studies indicating a stronger correlation between the CDR‐SB and observer (care partner)‐reported outcomes of daily function (eg, Functional Activities Questionnaire) compared with cognitive performance‐based measures (eg, ADAS‐Cog).^21^ Therefore, conceptually, we consider the daily activities anchor to be more aligned with the CDR‐SB. Second, changes in ADLs may be more readily observable and reliably reported by care partners, while memory changes may be more noticeable and robustly quantifiable through cognitive assessments performed by trained clinicians and neurologists. Of note, estimates for the memory anchor were similar, with slightly greater support for the lower end of the threshold range. Distribution‐based estimates for meaningful change on the CDR‐SB were generally less than a 1‐point change, further highlighting the importance of including care partner perspectives rather than statistical approaches alone when determining MWPC thresholds.

A 1‐point change, previously established as an appropriate threshold for MCI‐AD using clinician‐rated anchors,^10^, ^11^ was more readily associated with the “no change” category, suggesting a higher threshold (1.5 to 2 points) may be required to reflect meaningful deterioration that is notable to the care partner in our study cohort, which included participants at later stages of MCI (eg, closer to mild dementia) evaluated over a longer period. These findings reinforce the importance of using an appropriate threshold range that is calibrated to the target population when applying within‐patient thresholds to support the interpretation of treatment efficacy in a given clinical trial at a specific time point. For example, studies targeting earlier stages of MCI‐AD could still consider a 1‐point threshold, which potentially captures small yet meaningful changes in progression for this patient population. Furthermore, in studies of MCI‐AD over shorter durations, where less decline is expected across arms during the trial period, it may again be appropriate to select thresholds at the lower end of the proposed range.

MWPC estimates for the CDR‐SB based on the “somewhat worse” category on the CaGI‐Alz generally increased with time. This reflects one of the challenges associated with comparing a categorical anchor variable with a continuous outcome measure over time; an individual may persistently fall into the “somewhat worse” category over multiple time points but continue to deteriorate on the continuous CDR‐SB scale. However, the later time points demonstrated advantages when seeking a robust meaningful change threshold; stronger anchor‐measure correlations combined with greater separation between categories (Figure 1 and Table S1) provide greater certainty that individuals showing a change as rated on the CaGI‐Alz are having a meaningful change on the CDR‐SB (ie, there is less overlap of anchor categories and better characterization of change on the target measure). Over extended periods of time, the CDR‐SB change associated with each category on the anchor of interest may increase and be more susceptible to response shift/recall bias, with earlier time points perhaps being preferable to detect truly minimal amounts of change on the measure of interest.^29^ Our proposed range takes into account the relative strengths of both time points, providing an upper and lower bound for consideration in early AD populations, depending on the intended application.

In clinical trial populations enriched for faster progression, most participants will experience a numerical deterioration (Figure 4A,B), even in the context of an efficacious treatment meeting the effect sizes observed in recent trials of disease‐modifying therapies, as demonstrated in the 30% relative reduction simulation (Figure 4C,D). Here, eCDF plots can be useful to further contextualize the impact of treatment on patient progression; the simulated treatment arm is shifted to the left of the simulated placebo arm, indicating that patients in the simulated treatment arm are less likely to experience greater levels of progression on the CDR‐SB at both time points. This is further illustrated in the table insert, reporting the proportion of patients progressing by the proposed thresholds across arms. Minimal separation of the curves was observed at the lower end of our proposed threshold range, particularly at 73 weeks, suggesting that these estimates may be best applied in shorter trials. This highlights the importance of selecting MWPC estimates that are appropriate for the length of the study. The lower end of the proposed range may be more suitable to define meaningful progression over shorter trials, while the higher end of the proposed range may be more appropriate for longer trials where a larger proportion of the enrolled population may be expected to experience greater degrees of decline on the CDR‐SB. Overall, the simulation demonstrated a difference in the proportion of progressors for a simulated treatment benefit at the currently accepted relative reduction, thereby supporting their utility in interpreting treatment benefit.

This study has several limitations. First, while care partner reports are critical to understanding what constitutes a meaningful deterioration for people with AD, particularly with increased disease severity and loss of patient insight, there may be increased variability associated with their subjective judgments, especially in the absence of any formal informant training in how to respond to CaGI‐Alz items. Changes in care partners may also impact variability. The longitudinal collection of informant characteristics/changes was available for the majority of, but not all, participants. Within this subgroup, <5% of participants experienced a change in informant. Second, the CaGI‐Alz is a study‐specific global impression of change, which may be susceptible to recall bias and response shift over longer periods, which may impact the accuracy of reporting at the later time points.^29^ Furthermore, the psychometric properties of the CaGI‐Alz have not been well established, and while the item response structure is consistent with widely used global impression scales, the items could benefit from cognitive debriefing to ensure consistency of interpretation across raters. Cognitive debriefing of item responses can also be useful to support the meaningfulness of the anchor category selected for meaningful change analyses (in this case, the “somewhat worse” category). Third, overlapping confidence intervals for “no change” and “somewhat worse” groups in MCI‐AD at Week 49 for memory and mAD at Week 73 for daily activities could indicate a lack of ability to differentiate between these categories at the given time point. However, both eCDF plots and correlation analyses were supportive of anchor adequacy across time points, anchors, and subgroups. Fourth, there may be smaller changes that occur at an individual level on the CDR‐SB that are perceived to be meaningful to an individual patient but may not trigger a change on the CaGI‐Alz; our intent was to establish a range of MWPC thresholds using anchor‐based methods that reflect most patients, to support the interpretation of clinical trial results. Thresholds are based on a single clinical trial cohort and may not be generalizable to other patient cohorts of varying AD severities. Furthermore, the limited racial and ethnic diversity among trial participants may restrict the generalizability of our findings. Finally, qualitative research may further elucidate which aspects of impaired abilities lead care partners to rate the presence/absence of deterioration. This paper presents one proposed approach to evaluate treatment benefits and support the interpretation of clinical trial results, recognizing that there are a variety of ways that clinical meaningfulness can be further explored.

## 5 CONCLUSION

Although thresholds for MWPC may differ across disease stages, AD is a continuum, and the transition from MCI‐AD to mAD is seamless and indistinct. Applying a range of thresholds to clinical trial data is aligned with US Food and Drug Administration guidance and may be useful to support the interpretation of treatment benefits by enabling MWPC analyses. Our findings suggest that 1.5 to 2.5 points is an appropriate range to define meaningful within‐patient progression on the CDR‐SB in biomarker‐confirmed early AD populations analyzed over similar time periods. Importantly, MWPC analyses should apply thresholds that are calibrated to the context of use; lower/higher thresholds may be applicable in studies of earlier/later disease stages over shorter/longer durations.

## CONFLICT OF INTEREST STATEMENT

J.C., P.D., and C.J.L. are employees of F. Hoffmann‐La Roche Ltd and own shares in F. Hoffmann‐La Roche Ltd. E.T. and F.M. are employees of Genentech, Inc., part of F. Hoffmann‐La Roche Ltd, and own shares in F. Hoffmann‐La Roche Ltd. E.T. has patents for methods of treating neurodegenerative disease, publication nos. 2020131255 (30‐Apr‐2020) and 20210284720 (16‐Sep‐2021). R.P. and J.S. are employees of Roche Products Ltd and own shares in F. Hoffmann‐La Roche Ltd. J.L.C. has provided consultation to Acadia, Actinogen, Acumen, AlphaCognition, ALZpath, Aprinoia, AriBio, Artery, Biogen, Biohaven, BioVie, BioXcel, Bristol Myers Squibb, Cassava, Cerecin, Diadem, Eisai, GAP Foundation, GemVax, Janssen, Jocasta, Karuna, Lighthouse, Lilly, Lundbeck, LSP/eqt, Merck, NervGen, New Amsterdam, Novo Nordisk, Oligomerix, ONO, Optoceutics, Otsuka, Oxford Brain Diagnostics, Prothena, ReMYND, Roche, Sage Therapeutics, Signant Health, Simcere, Sinaptica, Suven, TrueBinding, Vaxxinity, and Wren pharmaceutical, assessment, and investment companies. J.L.C. owns the copyright of the Neuropsychiatric Inventory. J.L.C. has stocks/options in Artery, Vaxxinity, Behrens, Alzheon, MedAvante‐Prophase, and Acumen. J.L.C. has received the following grants: NIGMS grant P20GM109025; NINDS grant U01NS093334; NIA grant R01AG053798; NIA grant P30AG072959; NIA grant R35AG71476; NIA R25 AG083721‐01; ADDF; Ted and Maria Quirk Endowment; Joy Chambers‐Grundy Endowment. Author disclosures are available in the supporting information.

## CONSENT STATEMENT

The study protocols were approved by Institutional Review Boards prior to patient recruitment and conducted in accordance with the International Conference on Harmonisation E6 Guidelines for Good Clinical Practice. The study was carried out according to the laws of the country in which the trial was conducted. Each patient provided signed informed consent prior to study enrollment.

## Supporting information

Supporting Information

Supporting Information

## Data Availability

For eligible studies, qualified researchers may request access to individual patient‐level clinical data through a data request platform. At the time of writing, this request platform is Vivli: https://vivli.org/ourmember/roche/. For up‐to‐date details on Roche's Global Policy on the Sharing of Clinical Information and how to request access to related clinical study documents, see https://go.roche.com/data_sharing. Anonymized records for individual patients across more than one data source external to Roche cannot, and should not, be linked due to a potential increase in risk of patient re‐identification.
