# Supplementary material for: Care partner‐informed meaningful change thresholds for the Clinical Dementia Rating‐Sum of Boxes for trials of early Alzheimer's disease
Source: Alzheimers Dement. 2024 Jul 17;20(9):5889–900. doi: 10.1002/alz.14050 (PMC11497679; doi:10.1002/alz.14050)
Supplement: Supplementary file 2 — Supporting Information [file ALZ-20-5889-s002.docx]

**Supplementary materials for: Care partner-informed meaningful change thresholds for the CDR-SB for trials of early AD**

**Contents**

[**Supplementary Figure 1** Overview of the Caregiver Global Impression of Change – Alzheimer’s Disease 2](#_Toc157516624)

[**Supplementary Figure 2** Empirical cumulative distribution function plots of Clinical Dementia Rating Scale – Sum of Boxes change by Caregiver Global Impression of Change – Alzheimer’s Disease categories for mild cognitive impairment due to Alzheimer’s disease population 3](#_Toc157516625)

[**Supplementary Figure 3** Empirical cumulative distribution function plots of Clinical Dementia Rating Scale – Sum of Boxes change by Caregiver Global Impression of Change – Alzheimer’s Disease categories for mild Alzheimer’s disease dementia population 6](#_Toc157516626)

[**Supplementary Table 1** Correlation between anchor items at Weeks 25, 49, and 73 and change from baseline to Weeks 25, 49, and 72 in CDR-SB score 9](#_Toc157516627)


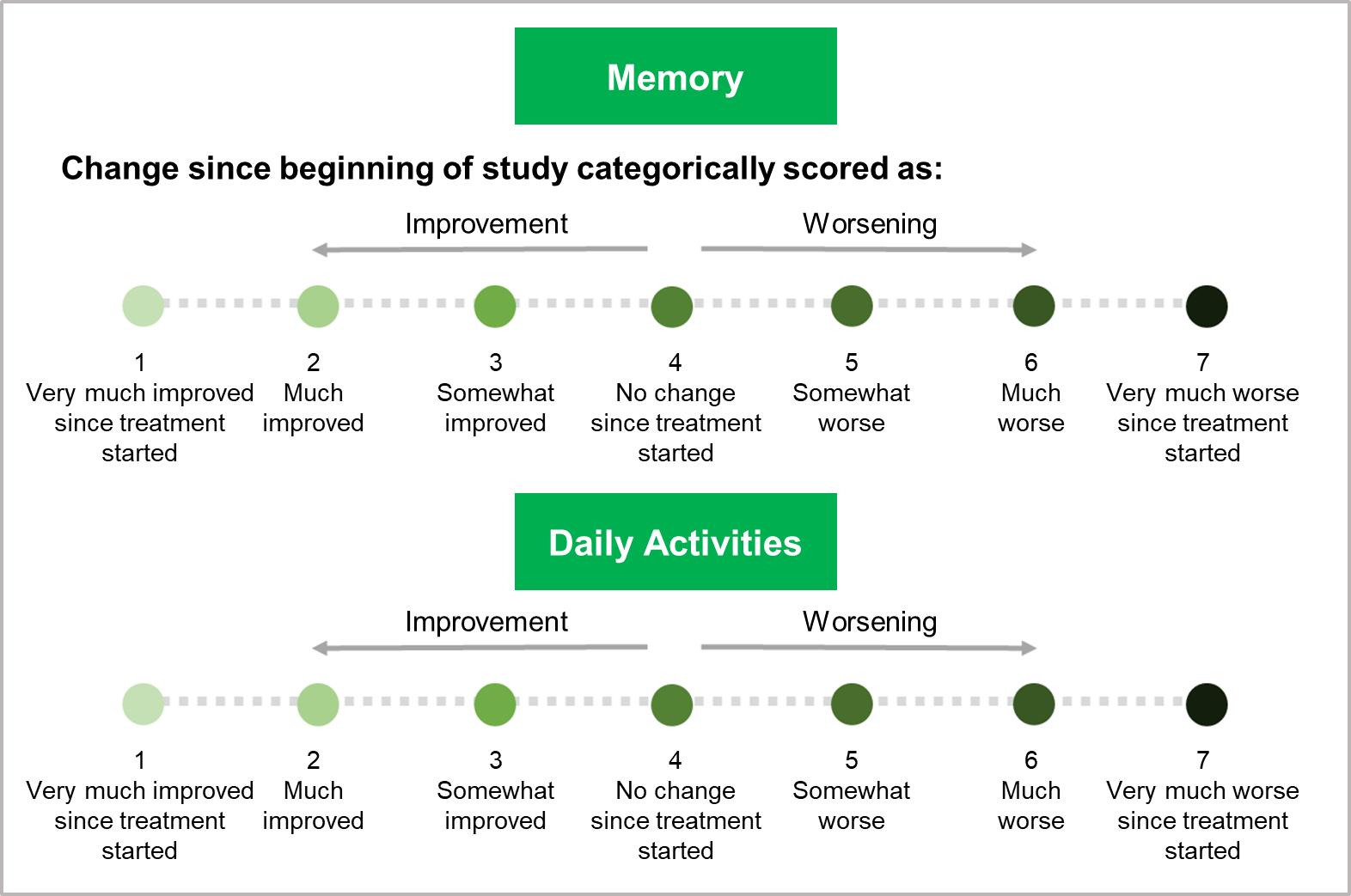


**Supplementary Figure 1** Overview of the Caregiver Global Impression of Change – Alzheimer’s Disease.


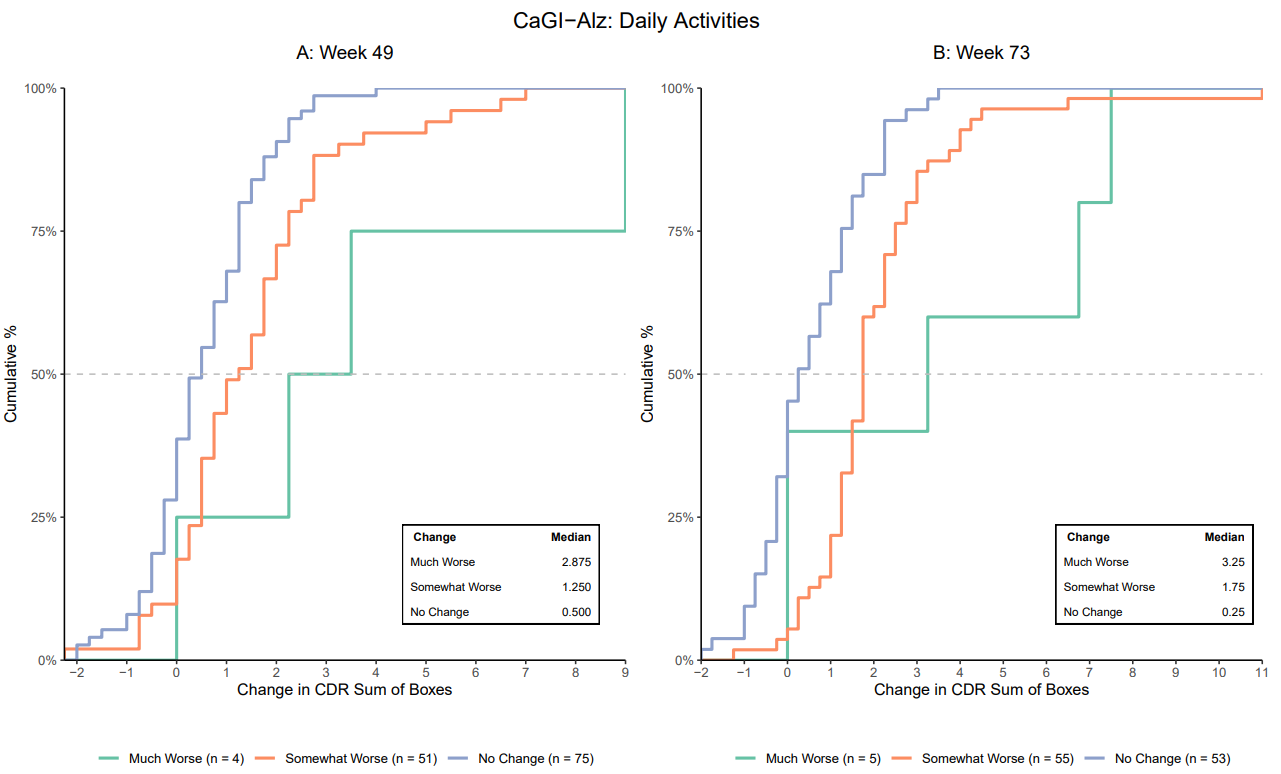


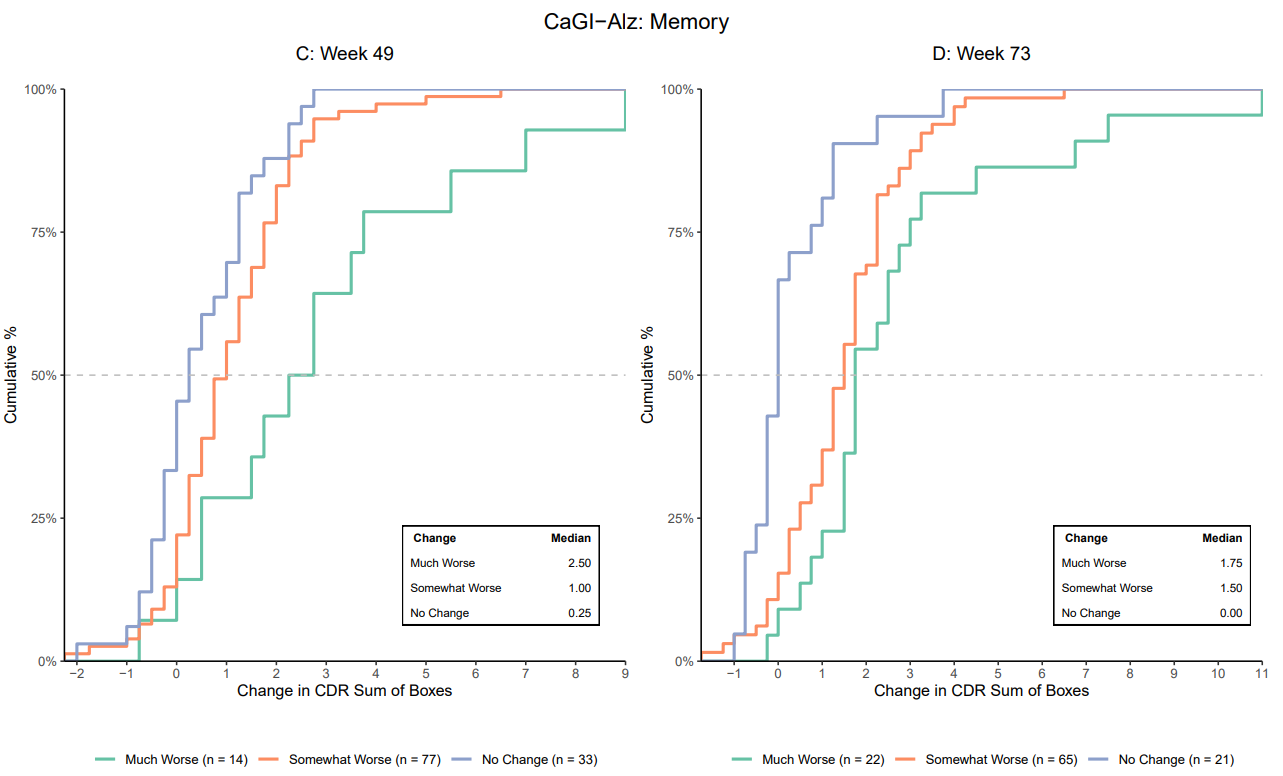


**Supplementary Figure 2** Empirical cumulative distribution function plots of Clinical Dementia Rating Scale – Sum of Boxes change by Caregiver Global Impression of Change – Alzheimer’s Disease categories for mild cognitive impairment due to Alzheimer’s disease population.

NOTE. eCDF plots display the cumulative percentage of individuals experiencing a given change on the CDR-SB from baseline to Weeks 49 and 73, respectively, grouped into "no change", "somewhat worse" or "much worse" on the CaGI-Alz Daily Activities (A and B) and CaGI-Alz Memory (C and D), respectively. “Improved” category not included in panels of eCDF plots due to low N (31), A: Week 49 (n = 6, median = 0.625); B: Week 73 (n = 4, median = 0.125); C: Week 49 (n = 12, median = –0.25); D: Week 73 (n = 9, median = 0).

CaGI-Alz, Caregiver Global Impression of Change – Alzheimer’s Disease; CDR, Clinical Dementia Rating Scale; eCDF, empirical cumulative distribution function; MCI-AD, mild cognitive impairment due to Alzheimer’s disease.


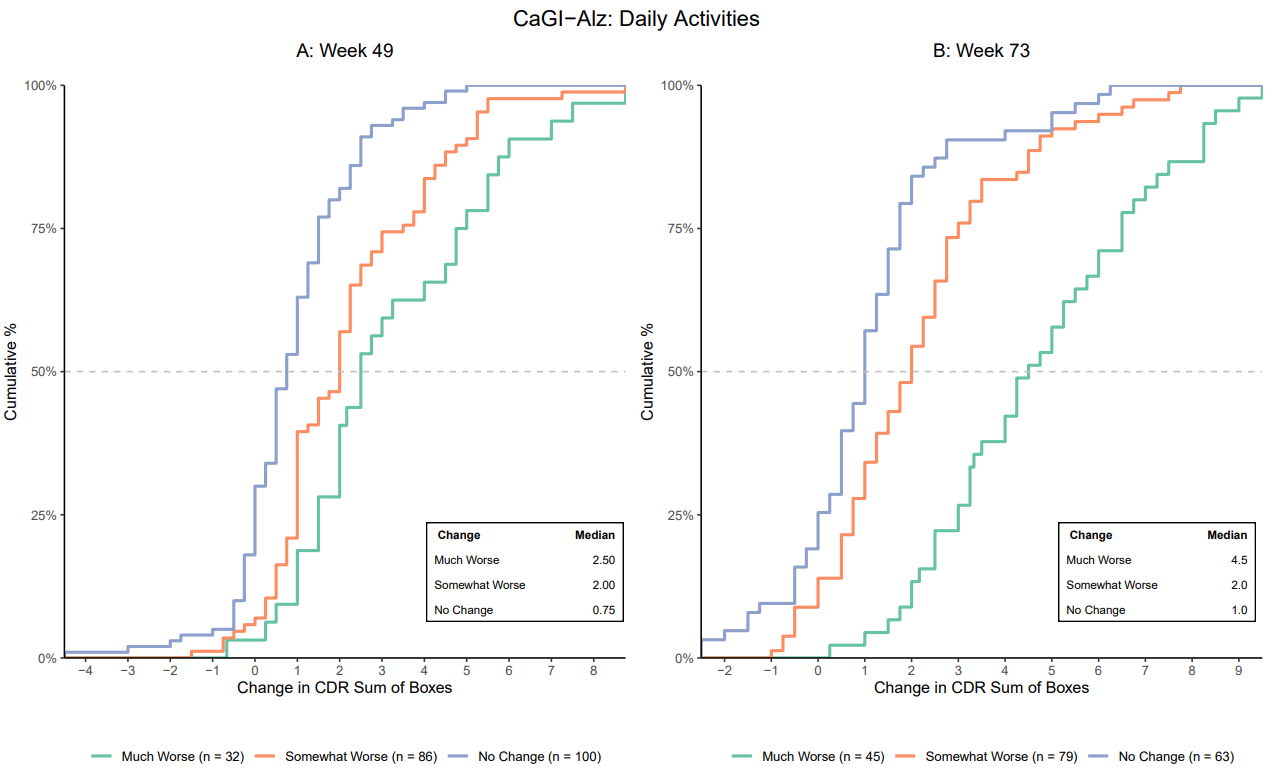

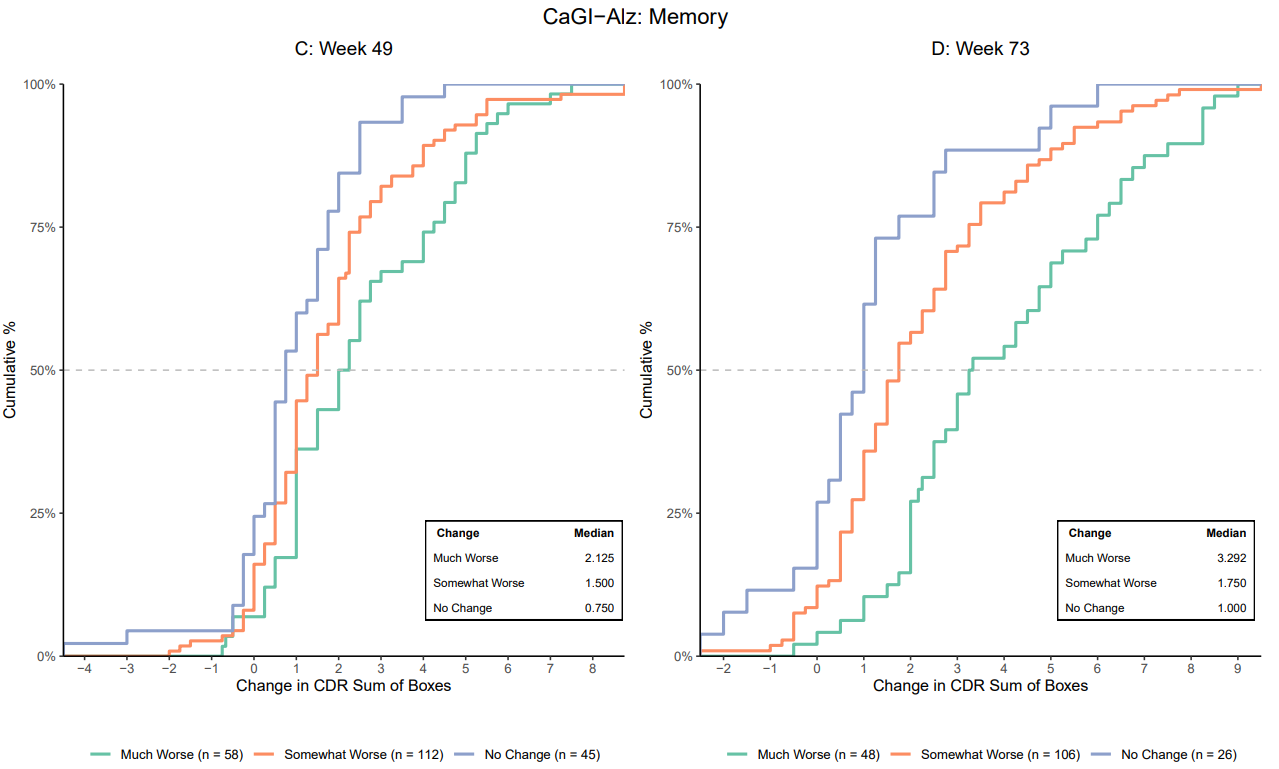


**Supplementary Figure 3** Empirical cumulative distribution function plots of Clinical Dementia Rating Scale – Sum of Boxes change by Caregiver Global Impression of Change – Alzheimer’s Disease categories for mild Alzheimer’s disease dementia population.

NOTE. eCDF plots display the cumulative percentage of individuals experiencing a given change on the CDR-SB from baseline to week 49 and 73, respectively, grouped into "no change", "somewhat worse" or "much worse" on the CaGI-Alz Daily Activities (A and B) and CaGI-Alz Memory (C and D), respectively. “Improved” category not included in panels of eCDF plots due to low N (40), A: Week 49 (n = 9, median = 1.5); B: Week 73 (n = 6, median = 2); C: Week 49 (n = 12, median = 0.125); D: Week 73 (n = 13, median = 0.5).

CaGI-Alz, Caregiver Global Impression of Change – Alzheimer’s Disease; CDR, Clinical Dementia Rating Scale; eCDF, empirical cumulative distribution function.

**Supplementary Table 1** Correlation between anchor items at Weeks 25, 49, and 73 and change from baseline to Weeks 25, 49, and 72 in CDR-SB score

|  | Early AD | | MCI-AD | | mAD | |
| --- | --- | --- | --- | --- | --- | --- |
| Timepoint | CDR-SB versus | | CDR-SB versus | | CDR-SB versus | |
|  | ADL anchor | Memory anchor | ADL anchor | Memory anchor | ADL Anchor | Memory anchor |
| Week 25 | 0.34 | 0.27 | 0.33 | 0.34 | 0.34 | 0.22 |
| Week 49 | 0.42 | 0.36 | 0.36 | 0.41 | 0.42 | 0.31 |
| Week 73 | 0.52 | 0.42 | 0.46 | 0.45 | 0.50 | 0.41 |

AD, Alzheimer’s disease; ADL, activities of daily living; CDR-SB, Clinical Dementia Rating – Sum of Boxes; mAD, mild Alzheimer’s disease dementia; MCI-AD, mild cognitive impairment due to Alzheimer’s disease.
